# Digital health understanding and preparedness of medical students: a cross-sectional study

**DOI:** 10.1080/10872981.2022.2114851

**Published:** 2022-08-28

**Authors:** Martin Baumgartner, Christoph Sauer, Kathrin Blagec, Georg Dorffner

**Affiliations:** Center for Medical Statistics, Informatics and Intelligent Systems, Medical University of Vienna, Vienna, Austria

**Keywords:** Digital health, eHealth, medical education, digital skills, curriculum

## Abstract

Digitalisation is changing all areas of our daily life. This changing environment requires new competences from physicians in all specialities. This study systematically surveyed the knowledge, attitude, and interests of medical students. These results will help further develop the medical curriculum, as well as increase our understanding of future physicians by other healthcare market players. A web-based survey consisting of four sections was developed: Section one queried demographic data, section two assessed the current digital health knowledge of medical students, section three queried their attitudes about the future impact of digital health in medicine and section four assessed the recommendations medical students have for the medical curriculum in terms of digital health. This survey was distributed to all (11,978) student at all public Austrian medical schools. A total of 8.4% of the medical student population started the survey. At the knowledge self-assessment section, the medical students reached mean of 11.74 points (SD 4.42) out of a possible maximum of 32 (female mean 10.66/ SD 3.87, male mean 13.34/SD 4.50). The attitude section showed that students see digitalisation as a threat, especially with respect to the patient–physician relationship. The curriculum recommendation section showed a high interest for topics related to AI, a per study year increasing interest in impact of digital health in communication, as well as a decreasing interest in robotic related topics. The attitude towards digital health can be described as sceptical. To ensure that future physicians keep pace with this development and fulfil their responsibility towards the society, medical schools need to be more proactive to foster the understanding of medical students that digital health will persistently alter the medical practice.

## Introduction

Digitalisation in medicine has existed for many years. In 2005 Austria introduced the e-card to authenticate patients via the Internet; the rollout for a nation-wide electronic health data record (ELGA) started in 2015. Internationally, telemedicine rose by 70% during the first year of the COVID-19 pandemic. In 2018, Apple obtained U.S. Health Authority (FDA) clearances for their Apple watch with electrocardiogram (ECG) and irregular rhythm notification functions. Almost every day a new decision support system using artificial intelligence algorithms is introduced. Digital health can also help to address staffing shortages, which is one of the top priorities of healthcare executives [1,2]. As a result, the World Health Organization (WHO) and consequently the European Union placed digital health as a top priority within their digital agenda [3,4].

To assure that future physicians can work with, contribute to and shape the development of digitalisation in medicine the medical education system is forced to provide the necessary digital training [5]. This means the need for new priorities to be included in an already packed medical curriculum. To address the definition and implementation of digital trainings across all Austrian public medical schools the government funded the project ‘Digital skills, knowledge and communication for medical curriculum’. This paper adds a comprehensive description of the medical student population of Austria by analysing the existing student’s digital health knowledge, the attitude towards digital health and their curriculum recommendations across the different years of study, including gender-specific deviations. This will allow a precise mapping of digital knowledge onto the existing curriculum.

A comprehensive, but non-systematic, literature review was performed. Existing literature describes the attitude and perception of medical students towards digital health [6–11], surveys identify recommended general content for digital health lectures [12–17], and teaching experience with digital health modules [18–20]. But there is no description of the medical student population as a whole. A questionnaire was developed to collect demographic data, data about knowledge, attitude and interests of medical students. The questionnaire was distributed via email and presented via web survey to all students at all public medical schools in Austria. Each section was separately analysed. Medical degree programs in the German-speaking countries last for 6 years and require a university entrance qualification.

## Methods

### Ethics approval and consent to participate

The data security board of the Medical University Vienna approved this cross-sectional study. The ethics committee of the Medical University Vienna waived the requirement for their decision. Participation was voluntary. At the first page of the survey, participants declared their consent.

### Inclusion criteria and recruitment

Registered medical students (year one to year six) at all public medical schools in Austria were eligible. Due to COVID-19 restricitions all lectures at the time of the study were performed via distance learning, therefore and in-line with the data protection rules students were invited by the student administration office of their relevant medical school. The student administration office sent out an invitation email including the survey link once by email to all medical students registered at three out of four medical schools (Medical University Vienna, the Medical University Innsbruck and the medical department of the Johann Kepler University Linz). The fourth medical school (Medical University Graz) provided the invitation at a web-based student message board. Recruitment took place from 26 November 2020, until 14 February 2021. No reminders were sent out.

### Study design

The aim of the study was to provide a comprehensive dataset about self-assessed knowledge, attitude and recommendations concerning the curriculum by medical students in the area of digital health. The study and survey were written in German, in line with the fact that the medical curricula at Austrian universities are taught in German. Questions were translated and shortened for this paper. The nature of the topics covered within the survey were medical databases, telemedicine, digital diagnostics, robotics, augmented & virtual reality, bio-signals and imaging, data security and digital communication. Following literature research, the survey was newly developed to satisfy the specific requirements of the study. It was deployed as a web-based survey using the SociSurvey platform (SW Version 3.2.11, SoSci Survey GmbH, München). At the time the survey was active all Austrian Universities relied on distance learning due to the COVID-19 restrictions. The web-based survey was chosen to be accessible by all students. The survey covers 4 areas: demographics, self-assessed knowledge of the student, attitudes of the students for the future impact of digital medicine and recommendation for the curriculum. In total 52 questions were defined.

The first section of demographic data consisted of six questions, covering university, age, gender, year of study and previous education. There were no points awarded in this section.

The second section ‘self-assessment’ consisted of 32 questions. For eight out of these 32 questions the student needed to select the right answer from several options. For 20 questions of the self-assessment of students were in the format of ‘can you explain the follow expression … Yes/No’. The Yes option was accompanied with an optional text field where the student could explain the expression. The explanations provided were used to verify the self-assessment of the students. Three questions were designed as symmetric five-level Likert items. A maximum of 32 points could be reached.

The third section ‘attitude’ consisted of 11 questions; one question with three options and 10 symmetric five-level Likert items, five of them with four 5-level Likert item sub-questions. The first question ‘how will digitalisation impact the patient–physician relationship’ ranged from 1 = ‘will be much worse’ to 5 = ‘will be much better’. The second question ‘how will digitalisation impact patient security within the operations theatre’ ranged from 1 = ‘patient security will be much lower’ to 5 = ‘will be much higher’. The remaining four questions covering ‘data protection’, ‘digital communication guidelines’, ‘ethic principles for digital communication’ and ‘handling of fake news’ ranged from 1 = ‘fully disagree’ to 5 = ‘fully agree’ and a sliding scale ‘relevance’ ranging from 1 to 101. For all Likert items the respective attitude was more positive towards digitalisation if the value was above three.

The fourth section ‘curriculum recommendation’ consisted of three questions. Students were asked to recommend topics for the curriculum. At each question the medical student received a list of ten predefined topics and the medical student had to select the three most preferred topics.

### Questionnaire

**Appendix Figure A1:**
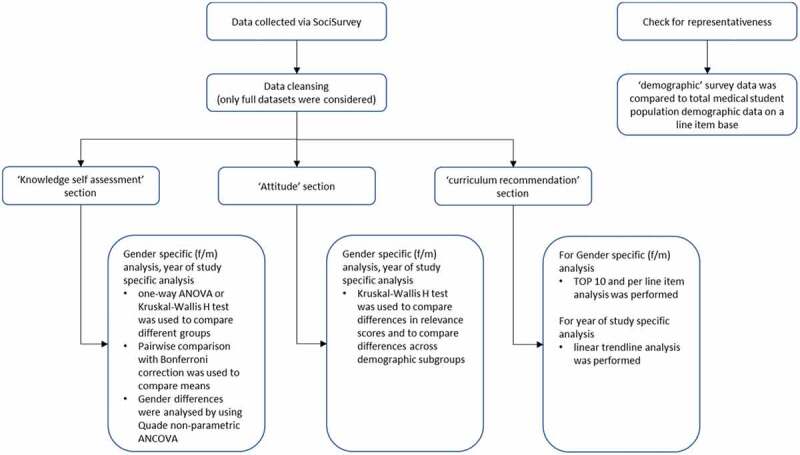
questionnaire design process.

To find an appropriate questionnaire (a flow chart for the questionnaire development process is shown in Appendix Figure A1) a literature review was performed. Databases including PubMed, Science Direct, and Google Scholar were searched. Keywords as ‘digital health’, ‘eHealth’, ‘dHealth’, ‘digital literacy’, ‘digitalisation’, ‘digital skills’ were combined with ‘medical curriculum’, ‘medical education’ and ‘medical students’.

The questionnaire is based on two pre-existing questionnaires [6,10]. Questions for the knowledge self assessment sections were collected from internal experts and lectures from courses for medical informatics and medicine at the university. In an internal review process, three researcher identified overlaps, discussed scales and agreed the questions.

The recruiting for the pre-test was done via social media and personal contacts, the questionnaire was provided via a web link. Sixteen students from non-medical courses filled out the questionnaire and comment on clarity, usability and general topics. Within the internal review process the feedback was discussed and agreed by three researcher, and incorporated into the questionnaire.

### Data collection

The data collection was anonymous; no data to identify the participating students was collected. The email invitation was sent out to 4,750 registered students at the Medical University Vienna, 3,468 registered students at the Medical University Innsbruck, 908 registered students at the Johannes Kepler University Linz and was presented to 2,852 registered students via an internet student message board at the Medical University Graz. In total 11,978 registered students were addressed. Via an embedded email link, the students were transferred to the first page of the survey at the SociSurvey webpage. At the first page they declared consent and then they could start the survey.

### Tests

Interval scales (section two) were tested for normal distribution with a Shapiro–Wilk test followed by a one-way ANOVA (normal distribution) or a Kruskal Wallis H Test (non-normal distribution).

Ordinal scales (section three) were tested for relevance scores with a Kruskal–Wallis H test.

### Data analysis

**Appendix Figure A2:**
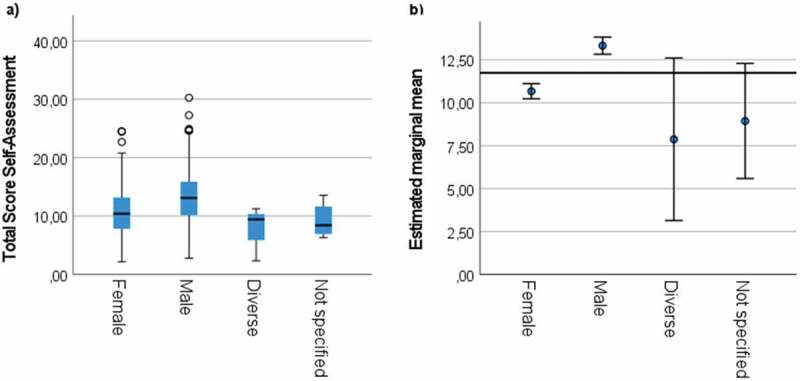
data analysis process.

The analysis was performed by questionnaire sections (a flow chart for the data analysis process is shown in Appendix Figure A2). Only fully performed questionnaires were included. Within each section, the results for female and male medical students were compared and the differences between different study years were calculated. To analyse if the medical students who participated in the survey are representative for the total medical student population the data from section one ‘demographics’ was used to compare the profile of participating medical students and the total medical student population.

Section two ‘self-assessment’: Depending on whether the assumption of a normal distribution of residuals (as determined by a Shapiro Wilk test) was met, either a one-way ANOVA or a Kruskal–Wallis H test (a Mann–Whitney *U* tests test in case of dichotomous variables) was performed to compare the differences between gender, study year, age group, ‘previous study or vocational training’ and educational background with regard to the self-assessment score. Pairwise comparison using Bonferroni correction was used to compare means between groups. To assess whether the differences in scores across ‘gender’ remain significant after controlling for age group, study year, ‘previous study or vocational training’ and educational background, a Quade non-parametric ANCOVA was conducted [21].

Section three ‘attitude’: In this section six 5-level-Likert items were analysed. For data presentation reasons five-level-Likert points one and two were combined to ‘disagree’ and five-point-Likert points four and five were combined to ‘agree’. A Kruskal–Wallis H test was used to compare differences in relevance scores with regard to gender and study year. For the question ‘how will digitalisation impact the patient–physician relationship’ a Kruskal–Wallis H test was used to compare differences across demographic subgroups.

Section four ‘curriculum recommendation’: To better understand the decision process itself the recommendation section was divided into three subsections. Within the different sub-sections different notions for the main topics (medical databases, telemedicine, digital diagnostic, robotic, augmented reality & virtual reality, bio signals and imaging, data security and digital communication) were used to better understand the ranking behaviour of the students. First the 10 most frequent recommended topics for the future curriculum development were determined. This top 10 list was then analysed for gender-specific differences. Furthermore, the data set was analysed for differences between female and male on a per-item base. Based on the overall female/male relation of the survey a trigger of +5% was defined to identify strong deviation. Finally, the curriculum recommendations were analysed for differences between different study years.

Statistical analyses were performed using SPSS version 28.0 (2021, IBM Corp Armonk, NY.) Microsoft Excel was used to prepare graphics.

## Results

### Demographics

The invitation to participate was sent out to 9,126 students by personal email, out of which 1001 responses were received (response rate 10.4%). 2,852 students were contacted via message board, out of which 68 responses were received (response rate 2.4%). The resulting overall response rate was 8.4%. Six hundred and fifty students finished the survey (55.3% female/43.0% male/1.7% others or undeclared), compared to the total medical student population 53.8% female/46.2% male. Table 1 shows the sociodemographic characteristics of the survey participants as compared to the total population of medical students. 91.4% reached qualification for the medical school at a high school with general focus, 4.6% reached it at a high school with technical curriculum, whereas one university did not provide this statistic. Within the total student population 95.4% reached qualification for the medical school at a high school with general focus. The number of participants per study year were 28.6% for year one, 18.6% for year two, 16.6% for year three, 13.1% for year four, 14.5% for year five and 8.0% for year six, respectively. 0.6% of the participants didn’t declare their study year. This differs from the distribution of all medical students (15.8% for year one, 17.1% for year two, 14.1% for year three, 14.1% for year four, 13.5% for year five and 25.5% for year six).

**Table 1.** Sociodemographic characteristics of the survey participants.

Sociodemographic characteristic	Survey Started		Survey completed		All Austrian medical students	
	*n*	%	*n*	%	*N*	%
Total	1017	100	650	100	11,978	100
Gender						
Female	575	56.5	357	54.9	6,446	53.8
Male	415	40.8	280	43.1	5,532	46.2
Diverse	3	0.3	3	0.5	N/A	N/A
Not specified	9	0.9	6	0.9	N/A	N/A
Missing	15	1.5	4	0.6	-	-
Age group						
<21	251	24.8	154	23.7	1,308^a^	11.8^a^
21–24	518	50.9	332	51.1	5,177^a^	46.8^a^
25–29	187	18.4	131	20.2	3,240^a^	29.3^a^
>29	39	3.9	24	3.7	1,345^a^	12.1^a^
Missing	22	2.2	9	1.4	-	-
University						
Medical University of Vienna	575	57.4	381	58.6	4,750	39.7
Medical University of Graz	68	6.8	39	6.0	2,852	23.8
Medical University of Innsbruck	196	19.6	125	19.2	3,468	29.0
Medical Faculty JKU Linz	163	16.3	101	15.5	908	7.6
Missing	15	1.5	4	0.6	-	-
Previous study or vocational training						
Yes	258	25.4	175	26.9	N/A	N/A
No	744	73.2	471	72.5	N/A	N/A
Missing	15	1.5	4	0.6	-	-
Educational background						
High school	833	81.8	551	84.8	8,122^§^	95.4^§^
School or training with technical focus	85	8.3	56	8.6	388^§^	4.6^§^
Other	84	8.3	40	6.2	N/A	N/A
Missing	15	1.5	3	0.5	-	-
Study year						
First	297	29.2	186	28.6	1,893	15.8
Second	197	19.4	121	18.6	2,045	17.1
Third	159	15.6	108	16.6	1,689	14.1
Fourth	135	13.3	85	13.1	1,690	14.1
Fifth	130	12.8	94	14.5	1,612	13.5
Sixth	83	8.2	52	8.0	3,050	25.5
Missing	16	1.6	4	0.6	-	.

^a^*Values do not include data from the Medical Faculty JKU Linz who was only able to provide aggregate statistics: Average age of students enrolled in bachelor studies: 22.5/master studies: 25.5*. ^§^*Values exclude data from the Medical University of Innsbruck who was unable to provide data for this characteristic.*

### Knowledge self-assessment

Survey section two ‘self-assessment’ consisted of 32 questions. For every question the participant could achieve one point, thus, a maximum of 32 points was theoretically achievable. Exemplary questions are ‘can you explain the differences between augmented reality and virtual reality’, ‘can you explain the term digital therapeutics’, ‘can you explain the difference between artificial intelligence and machine learning’, ‘can you explain the differences between supervised and unsupervised learning’, ‘can you explain the term Electronic Health Record’, ‘can you explain why medical standards as HL7 are needed’.

On average, students achieved 11.74 points (SD: 4.42). Shapiro–Wilk test and graphical inspection revealed that assumptions for one-way ANOVA (normal distribution of residuals) were not met. Therefore, Kruskal–Wallis H and Mann–Whitney U tests were used to compare differences between demographic subgroups. Descriptive statistics are shown per demographic subgroups and results of the Kruskal–Wallis H and Mann–Whitney *U* tests in Appendix Table S2. Results showed that there was a statistically significant difference in scores between at least two groups for all queried demographic variables.

**Appendix Figure A3:**
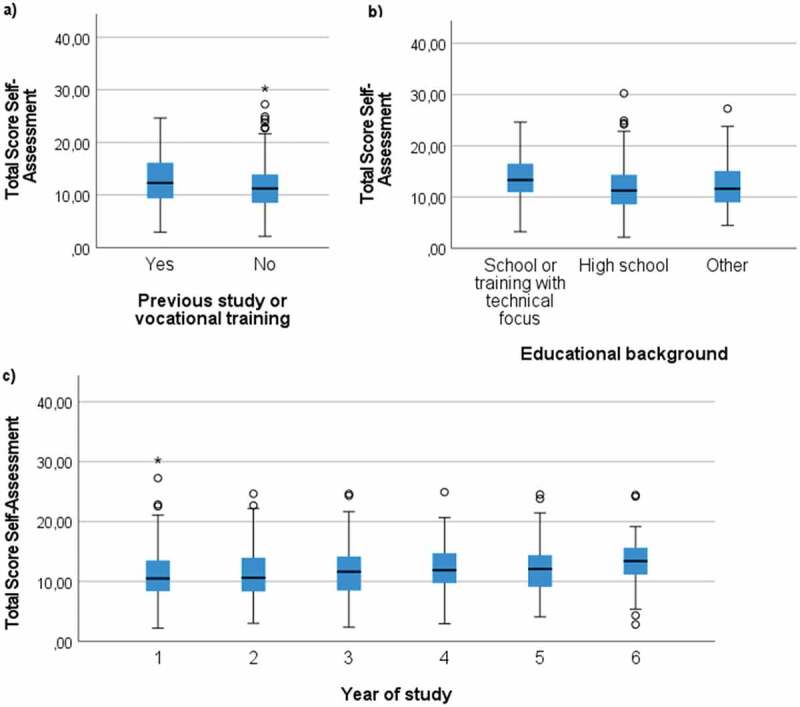
a) Student self-assessment score per gender, b) Estimated marginal means per gender after controlling for age group, study year, ‘previous study or vocational training’ and educational background. Whiskers in b) indicate marginal mean ± 2x standard error. Note the different y-axis range between a) and b).

**Appendix Figure A4:**
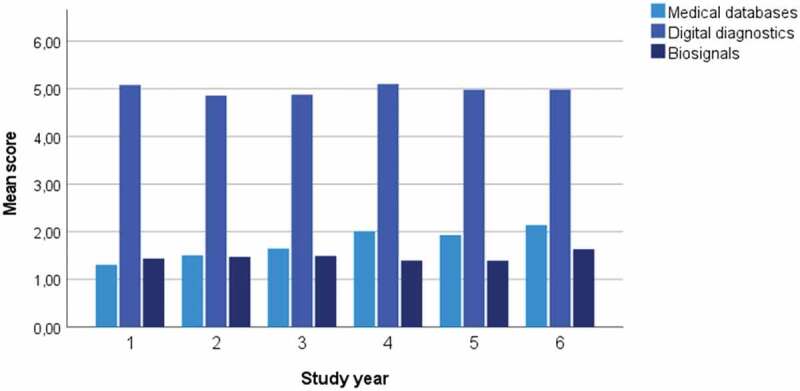
Student self-assessment score per a) ‘previous study or vocational training’, b) educational background and c) year of study.

In median, participants who identified as ‘male’ had significantly higher scores than those who identified as ‘female’ (p < .001, p_adj_<.001). Results showed that the difference in average self-assessment score between gender ‘male’ and ‘female’ remained significant with a t statistic (903 degrees of freedom) of −6.69 (*p* < .001) after controlling for age group, study year, ‘previous study or vocational training’ and educational background. Since results of parametric ANCOVA were comparable to non-parametric Quade’s ANCOVA, we additionally report estimated marginal means per gender. Further, multiple linear regression was used to test if study year, educational background and ‘previous study or vocational training’ significantly predicted self-assessment score. Unadjusted scores per gender and estimated marginal means after controlling for age group, study year, ‘previous study or vocational training’ and educational background, respectively, are shown in Appendix Figure A3a and A3b. Unadjusted scores for the demographic variables ‘previous study or vocational training’, educational background and year of study are shown in Appendix Figure A4. Participants who reported to have completed a school or training with technical focus had, in median, significantly higher scores than those who completed a high school (*p* = <.001, *p*_adj_ =_ _.001). Further, students who completed a previous study or vocational training had, in median, significantly higher scores than those without a previous study or training (*p* = .001). Multiple linear regression was used to test if study year, educational background and ‘previous study or vocational training’ significantly predicted self-assessment score. The model achieved an R2 value of 0.038, and it was found that both study year (β = 0.326, *p* = .001) and ‘previous study or vocational training’ (β = −1.506, *p* < .001) were statistically significant predictors. After adjusting for age as a potential confounding factor, ‘previous study or vocational training’ remained as the only significant predictor of self-assessment score in the adjusted model (β = −1.259, *p* = .005). The effect of study year diminished compared to the unadjusted analysis (β = .236, *p* = .069).

**Appendix Figure A5:**
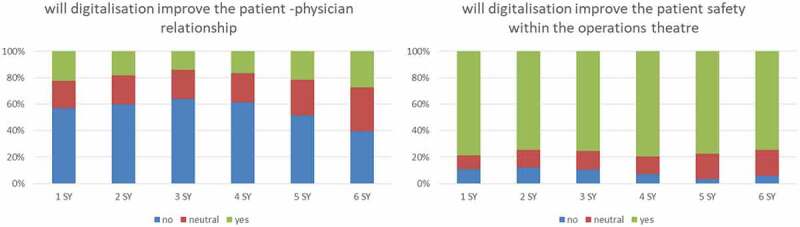
Student self-assessment score per topic area per study year.

For more comprehensive results the questions were clustered in 3 groups ‘digital diagnostics’ (10 questions), ‘bio signals’ (5 questions) and ‘medical databases’ (9 questions). For ‘digital diagnostics’ mean was 4.96/SD 1.82 (female mean 4.79/SD 1.73, male mean 4.06/SD 1.85), for ‘bio signals’ mean was 1.45/SD 1.01 (female mean 1.17/SD 0.86, male mean 0.64/SD 0.74) and for ‘medical databases’ mean was 1.64/SD 1.48 (female mean 1.32/SD 1.35, male mean 1.83/SD 1.07). The results per study year are shown in Appendix Figure A5. Medical students scored best in the area of ‘digital diagnostics’ with a mean value of approximately five points out of ten in all study years. For ‘bio signals’ they scored a mean value of close to 1.5 points out of five in all study years. In the area of ‘medical databases’ the mean value increased from 1.30 (study year one) to 2.14 (study year six).

### Attitudes and perceptions

Medical students assumed ‘digitalisation will improve patient safety within the operations theatre’ (median 4, agree 77.1%, disagree 8.9%), while males were more positive (median 4, agree 85.8%, disagree 5.1%) than females (median 4, agree 70.9%, disagree 11.9%).

Descriptive statistics for items that assessed attitudes and subjective relevance assessment of topics related to data protection, digital communication and fake news in medicine are shown in Appendix Table A3.

‘Data protection’ in general was seen as very important by students for their professional career (median 5, agree 89.1%, disagree 3.9%, female median 5, agree 90.2%, disagree 1.7%, male median 4, agree 87.5%, disagree 6.8%), sufficient coverage of data protection in existing lectures was deemed average with median 3, agree 34.7%, disagree 34.4% (female median 3, agree 31.7%, disagree 36.8%, male median 3, agree 38.6%, disagree 30.7%).

All communication topics related to the internet and digital channels, physician – patient communication as well as information research by the physician, received high attention by students. ‘Knowledge of digital communication guidelines’ was seen as relevant for their professional career (median 4, agree 69.4%, disagree 4.1%, female median 4, agree 70.8%, disagree 3.1%, male median 4, agree 67.3%, disagree 5.5%), but the topic was deemed to be not properly covered within the current curriculum (median 2, agree 31.8%, disagree 39.9%, female median 2, agree 30.4%, disagree 40.1%, male median 2, agree 34.5%, disagree 39.2%). ‘Knowledge of ethical principles for digital communication’ were rated as relevant for their professional career (median 4, agree 73.8%, disagree 3.3%). It was rated more relevant by females (median 4, agree 79.7%, disagree 1.4%) than by males (median 4, agree 66.3%, disagree 5.9%), whereas the coverage in the existing curriculum was rated insufficient by both males and females (median 2, agree 11.2%, disagree 59.3%, female median 2, agree 10.4%, disagree 60.8%, male median 2, agree 12.5%, disagree 57.0%).

For gender, the Kruskal–Wallis test revealed that there was a statistically significant difference in median scores between at least two groups with regard to the overall relevance ratings of the topics ‘data protection’ (H [1,3] = 10.28; p = .016), ‘knowledge of digital communication guidelines’ (H [1,3] = 10.03; p = .018) and ‘knowledge of ethical principles for digital communication’ (H [1,3] = 36.21; p = <.001). Pairwise comparison using Bonferroni correction showed that, in median, female students rated the relevance of ‘knowledge of digital communication guidelines’ (Mdn 78.92 [IQR = 100.25–66.00] vs. 73.15 [IQR = 91.00–59.75]; p = <.001, padj = <.001), ‘knowledge of ethical principles for digital communication’ (Mdn 85.64 [IQR = 101.00–77.00] vs. 77.49 [IQR = 101.00–65.00]; p = <.001, padj = <.001) and ‘data protection’ (Mdn 95.22 [IQR = 101.00–93.00] vs. 91.40 [IQR = 101.00–86.00]; p = .003, padj = .009) higher than male students.

Female and male medical students were equally concerned about ‘effective dealing with fake news’ for their professional career (median 4, agree 79.9%, disagree 5.3%, female median 4, agree 78.9%, disagree 3.9%, male 4, agree 81.5%, disagree 6.9%). Sufficient coverage of this topic in the existing curriculum was rated negative (median 2, agree 12.9%, disagree 59.9%, female median 2, agree 10.7%, disagree 62.8%, male median 2, agree 15.9%, disagree 56.7%). The question ‘will digitalisation improve the physician–patient communication’ was seen negatively (median 2, yes 19.7%, no 56.9%, female median 2, yes 17.8%, no 62.3%, male median 2, yes 21.5%, no 50.7%). The Kruskal–Wallis H test was used to compare differences between demographic subgroups. No statistically significant differences were found.

**Appendix Figure A6:**
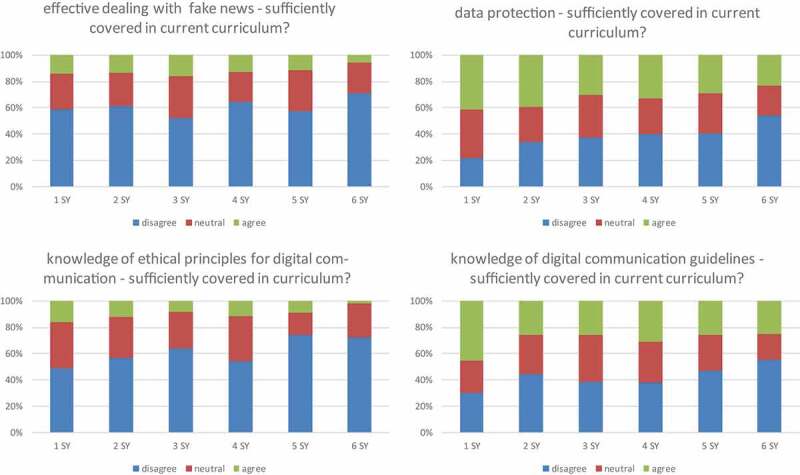
Attitudes towards impact of digitalization per study year.

The analysis per study year of ‘will digitalization improve the patient – physician relationship’ across the different study years (Appendix Figure A6) show in study year one to four close to 60% of the medical students of the specific study year marked ‘no’. In study year five and six the negative attitude decreased and in year six only 39.2% marked ‘no’. The analysis of ‘will digitalization improve the patient safety within the operations theatre’ (Appendix Figure A6) shows, close to 80% of medical students of all study years marked ‘yes’, meaning that digitalization will improve patient safety.

**Appendix Figure A7:**
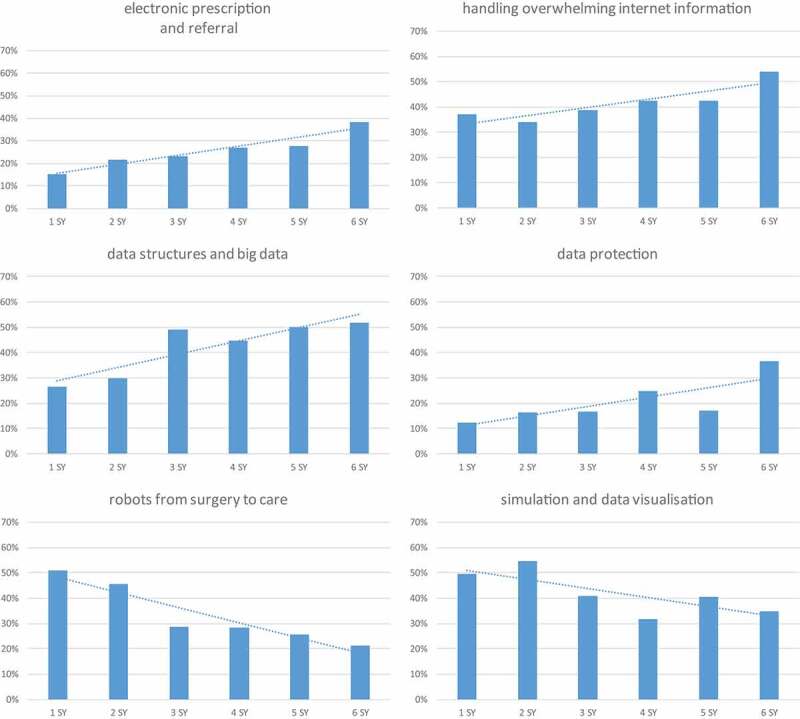
Satisfaction with digital communication trainings per study year.

As shown earlier, data protection is seen as important for their professional career by almost 90% of the medical students. Appendix Figure A7 shows that 41.1% of year one medical students are satisfied with their data protection training within the current curriculum. This value decreases consistently until study year six, in which only 23.1% of medical students are satisfied with data protection trainings. Also, more than 70% of all medical students see ‘knowledge of ethical principles for digital communication’ and ‘knowledge of digital communication guidelines’ as important for their professional career. The satisfaction with training for ‘knowledge of digital communication guidelines’ goes down from 45.4% of medical students in study year one to 25.0% of medical students in study year six. The satisfaction with training for ‘knowledge of ethical principles for digital communication’ goes down from 15.8% in year one to 2.0% in year six. ‘Effective dealing with fake news’ is seen by around 80% of all medical students as important for their professional career. 14.1% of all medical students in study year one is satisfied with trainings for ‘effective dealing with fake news’ compared to 5.8% in study year six.

### Curriculum recommendations

For the first qualitative analysis in section four ‘curriculum recommendation’ the responses were combined across all three questions to create a TOP10 list to identify the most recommended topics for the curriculum. It was identified that one recommendation (‘increasing knowledge on communication protocols’) was interpreted by the students in two different ways and it was therefore excluded from the analysis. The most recommended topics were 1) digital diagnostics, 2) artificial intelligence and machine learning, 3) patient self-diagnosis with apps and ‘Dr. Google’, 4) simulation and data visualisation, 5) decision support systems, 6) handling overwhelming internet information, 7) bio signals and bio signal processing, 8) data structures and big data, 9) robotics from surgery to care, and 10) ethical and legal aspects of digital communication channels. The TOP10 list analysed for female and male showed only minor differences. To understand if there are specific curriculum recommendations selected mainly from female or male medical students the data was analysed on a per recommendation base. The female/male relation for the survey overall is female 54.9%, male 43.1%, other 2.0%. A deviation of more than +5% was selected as relevant. The recommendations where the female proportion was 59.9% or higher were: ‘ethical and legal aspects of digital communication channels’ (64.1%), ‘imaging and imaging processing’ (62.7%), ‘support systems for surgeons’ (61.6%), and ‘storing and administration of patient data’ (60.7%). The recommendations where the male proportion was 48.1% or higher were; ‘augmented and virtual reality’ (53.2%), ‘text and speech recognition’ (50.0%), ‘wearables and internet of things’ (49.2%), ‘data structures and big data’ (48.6%), and ‘bio signals and bio signal processing’ (48.2%).

**Figure 1.**
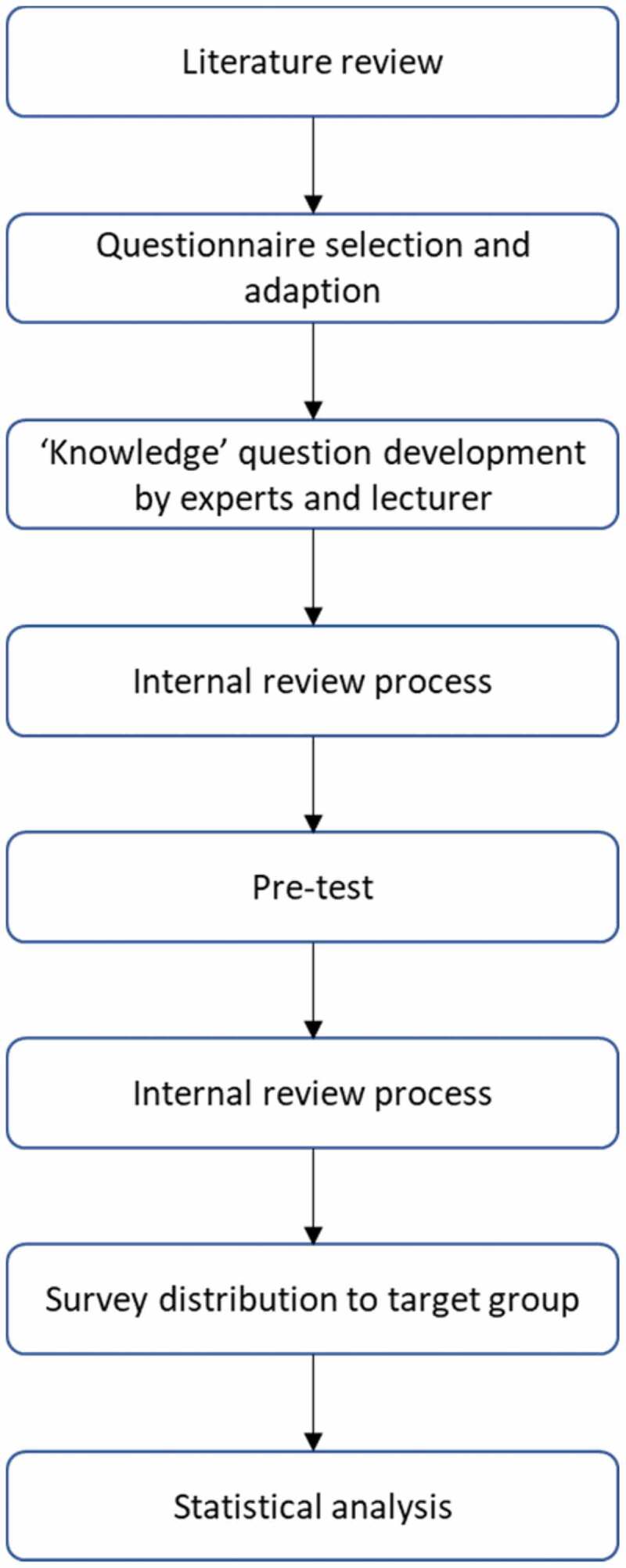
Trend line analysis for curriculum recommendations per study year.

To analyse changes in curriculum recommendations between study year one and study year six a linear trend line was used (Figure 1). The recommendations with the biggest increase are ‘electronic prescription and referral’, which was selected by 15.1% of the medical students in study year one and 38.5% of the medical students in study year six, ‘handling overwhelming internet information’ was selected by 37.1% in study year one and 53.8% in study year six, ‘data structures and big data’ was selected by 26.3% in study year one and 51.9% in study year six, ‘tele-consultation with patients or peers’ was selected by 15.1% in study year one and 32.7% in study year six, ‘data security’ was selected by 12.4% in study year one and 36.5% in study year six. The recommendations with the biggest decrease are ‘simulation and data visualisation’, it was selected by 49.5% of the medical students in study year one and 34.6% of the medical students in study year six, ‘support systems for surgeons’ were selected by 40.9% in study year one and 13.5% in study year six, ‘robots from surgery to care’ was selected by 51.1% in study year one and 21.2% in study year six.

## Discussion

Existing papers and European initiatives focus on content regarding digital health and on the curriculum implementation [22,23]. This paper adds a medical student-centred perspective to better understand the capabilities and limitations of medical students. The results of the survey give medical schools the opportunity to meet the medical students where they are in terms of knowledge, attitude, expectations and interests.

The survey shows medical students know on average only one-third of the digital terms queried in the survey. Most known terms are from the area of digital diagnostics, followed by medical databases and bio-signals and imaging [24]. The results are consistent over the different study years; this suggests that digital knowledge is not acquired as part of their studies; it is driven by articles from public sources such as digital news feeds and newspapers. These articles usually provide an easily understandable snapshot about a topic, but do not provide enough detail for an understanding of the underlying concepts. Due to the fact that the current curricula do not cover digital health in-depth, it was expected that medical students would acquire digital health knowledge in line with the development of their medical education. Within the investigated areas there is no knowledge increase from study year one to study year six, except a slight increase in the area of medical databases. As seen in many other subjects, students are reluctant to deal with science, technology, engineering and mathematics (STEM) content [25–27], this result is also valid for medical students. Therefore, it is far more than teaching medical students digital health; it is about stimulating interest in the medical students for digital technology and demonstrating the potential benefits [8,15,19,28,29].

Medical students are aware of the high potential of new digital health applications. They see positive results, such as an increase in patient safety due to an increased digitalisation in the operation theatre. Additionally, nearly all medical students see data protection as important or very important for their professional career. A third of medical students are satisfied with the current level of training for data protection. But only 18% of medical students recommend training for data protection; medical students leave it with the application developer and medical agencies to ensure high data protection. The results clearly show medical students are afraid about communication in the digital world. This is in line with the general finding from the SINUS study, which found that a common behaviour shift is ongoing where the past enthusiasm for the benefits of the Internet is overshadowed by the fear to lose control about privacy and personal data [30]. Nearly all medical students see patient-physician communication over digital channels and handling of fake news as important or very important for their professional career. Nevertheless, more than half of medical students expect a worsening of the patient-physician communication due to digital health. Consequently, two-thirds of all medical students request additional digital communication training, in addition to the existing face-to-face physician-patient communication training [11]. The results are independent of the digital health knowledge of the individual medical student. It shows a common reluctance towards the possibilities of digitalisation within communication [31].

Furthermore, the results show that medical students miss an overview about the many and various areas of digital health applications. Medical students see themselves more as digital health users than as digital health creators. When asked to recommend further training areas, medical students focus on digital diagnostics, which is very popular and covered by many public sources. Beyond digital diagnostics knowledge and interests significantly drop. Analysed based on study year, the interests of medical students on digital health technology decrease over time whereas the interests on communication via digital channels rises. The self-conception of Austrian medical students is focused on the importance of the face-to-face physician-patient communication [11]. Other countries, where an application as tele-health is common, have a much more positive attitude toward digital health applications [32]. Therefore, to fulfil the future requirements by the society medical students need to better understand the benefits as well as the limitations of digital health [19].

To provide medical students with a sound basic understanding of digital health it is recommended, based on the findings of this study, to implement digital health skills within the curriculum in the following way;

- Medical students at the beginning of their studies are more interested in digital health technology, therefore fundamentals and basic application concepts of digital health technologies need to be trained in parallel to biology and chemistry as part of the pre-clinical subjects.
- Existing training for medical statistics needs to be interwoven with clinical reasoning to develop data driven reasoning as further option for diagnosis [33–37].
- Concepts, benefits and limitation of state-of-the-art applications need to be taught as part of the respective medical specialty in the second part of their studies.
- At the end of their studies medical students are interested more in communication via digital channels. This interest should be used to incorporate telemedicine, ethics in social media communication and data security into the existing physician-patient communication training [28,29,38].
- Furthermore, to promote interest for both lecturer and students the benefits and opportunities of digital health it is recommended to organize open lectures to present outstanding digital scientific and medical digital health projects.

## Limitations

The number of students ranged from 908 (Johannes Kepler University Linz) to 4,750 registered students (Medical University Vienna). The number of responses ranged from 68 (Medical University Graz) to 575 (Medical University of Vienna). As the Medical University of Graz used a different way to approach the students, the representation of the different university is uneven. For further studies a better coordination of the approaches will reduce those differences.

The medical school at the Johannes Kepler University Linz began in 2014. They are still in their ramp-up phase, which on one hand leads to a response rate of 18.0%, but with a significant number of responses from one- to third-year students.

From the 1017 students that started the survey only 650 finished the survey. The reasons for the drop-out rate were discussed twofold. On one side, the survey consisted of 52 questions, which makes it a long web-based survey. On the other side, the mean at the self-assessment section were 11.78 (min 1.00, max 27.4) out of 31.97 – the questions could have been too difficult and the participants could have lost interest. For further surveys a survey optimization may help to reduce the number of dropouts.

To understand if the survey data is representative for the whole medical student population the size of a minimum random sample to satisfy a confidence interval of 95% and an error range of 5% was calculated with 373 survey responses. To investigate the potential bias of the survey student population the demographics were compared with the total student population. The gender split of the survey is female 55.3%, male 43.0%, compared to the total medical student population of 53.8%/46.2%. The participation rate across the different study years is uneven. 47.2% of survey participants come from study year one and two, whereas just 8.0% are coming from study year six. The number of students who earned their university qualification at a high school with technical curriculum was 8.6% among survey participants compared to 4.6% in the total student population. The higher number of students from high schools with technical curriculum will potentially lead to a more positive picture about existing digital health knowledge than the total population.

## Conclusions

Our study shows that despite the fact all current medical students have grown up in the digital world, it is not obvious that medical students are interested in and have a basic understanding of technology and especially digital health. Furthermore, the students are not entirely positive towards the opportunities of digital technology. As described in the SINUS study in Western Europe students in general use smart phones and are connected to the Internet. But the Internet is seen as commodity and mainly used as tool to communicate with peers [30,39].

It is clear that digital health will change health care; and only by providing medical students with a solid understanding of the basic concepts and capabilities of digital health, the future physicians will understand the options and benefits of digital health in their daily routine. This will give them the opportunity to be more open minded toward digital health and help reduce reluctance on the adoption of digital health. The current knowledge is driven by curiosity. This means, for example that applications using artificial intelligence are known, but seen as isolated tools. This approach will not generate reliable knowledge to critically evaluate and use upcoming digital health developments. Digital health also provides new approaches to diagnosis and therapy. By using the capabilities of medical databases digital health provides a new way of understanding diseases. To use these new capabilities, physicians need to add data driven thinking to contextual reasoning and intuition to fully benefit from the opportunities of medical databases [34,37,40].

The findings of this cross-sectional study provide a foundation for medical schools to develop the curriculum towards digital health in a student-centered way. Longitudinal studies are needed to prove the findings in actual curriculum mappings. Content about digitalization in medicine needs to come in line with the digital capabilities of medical schools. Therefore, medical schools need to develop their digital capabilities and learning environment. This shows a need for further research.

## Data Availability

The authors confirm that the data supporting the findings of this study are available within its supplementary materials.

## Appendix

**Appendix Table A2: Means, standard deviations, and Kruskal-Wallis*H*/Mann-Whitney*U* tests**

**Table:**

Variable	Self-assessment score^a^					
	*M*	*SD*	*Min*	*Max*	*H/MW-U*	*p*
Gender					*H*(1,3) = 63.53	<.001
Male	13.3	4.5	2.8	30.25		
Female	10.64	3.9	2.2	24.5		
Diverse	7.7	4.7	2.3	11.3		
Not specified	9.2	2.9	6.3	13.6		
Age group						
<21	10.6	4.1	2.2	30.3	*H*(1,3) = 22.72	
21–24	11.9	4.3	2.3	27.3		<.001
25–29	12.7	4.6	2.9	24.2		
>29	12.8	4.0	4.3	20.9		
Previous study or vocational training						
Yes	12.9	4.6	2.9	24.7	*MW-U *= 34,399.50	.001
No	11.4	4.2	2.2	30.3		
Educational background						
High School	11.5	4.3	2.2	30.3	*H*(1,2) = 12.52	.002
School or training with technical focus	13.8	4.6	3.3	24.7		
Other	12.3	4.8	4.5	27.3		
Study year						
First	11.3	4.4	2.2	30.3	*H*(1,5) = 14.71	.012
Second	11.4	4.5	3.0	24.7		
Third	11.8	4.5	2.3	24.6		
Fourth	12.3	4.2	2.9	24.9		
Fifth	12.0	4.0	4.1	24.5		
Sixth	13.2	4.3	2.8	24.4		

^a^Maximum reachable score: 31.97

**Appendix Table A3: Attitudes towards data protection, digital communication and fake news**.

**Table:**

	Data protection		Digital communication guidelines		Ethic principles for digital communication		Fake news	
	*Mdn*	*IQR*	*Mdn*	*IQR*	*Mdn*	*IQR*	*Mdn*	*IQR*
Has significantly impacted medicine during the last 5 years. ^a^	4	5–4	3	4–3	3	4–3	4	5–3
Will impact medicine during the next 5 years. ^a^	5	5–4	4	5–3	4	5–3	5	5–4
Is highly important for my future professional career. ^a^	5	5–4	4	5–3	4	5–3	4	5–4
Is not properly covered within the curriculum. ^a^	3	4–2	2	3–1	2	3–1	2	3–1
	*Mean*	*STD*	*Mean*	*STD*	*Mean*	*STD*	*Mean*	*STD*
Relevance ^b^	93.62	13.31	75.81	23.09	81.67	22.88	88.87	21.38
								

^a^Items were Likert scale items ranging from 0 – do not agree to 5 – strongly agree.

^b^Items were assessed on a sliding scale ranging from 0 to 101.
